# Location-Specific Comparison Between a 3D In-Stent Restenosis Model and Micro-CT and Histology Data from Porcine *In Vivo* Experiments

**DOI:** 10.1007/s13239-019-00431-4

**Published:** 2019-09-17

**Authors:** P. S. Zun, A. J. Narracott, C. Chiastra, J. Gunn, A. G. Hoekstra

**Affiliations:** 1 Institute for Informatics, Faculty of Science, University of Amsterdam, Amsterdam, The Netherlands; 2 Biomechanics Laboratory, Department of Biomedical Engineering, Erasmus Medical Center, Rotterdam, The Netherlands; 3 National Center for Cognitive Technologies, ITMO University, Saint Petersburg, Russia; 4 Department of Infection, Immunity & Cardiovascular Disease, University of Sheffield, Sheffield, UK; 5 Insigneo Institute for In Silico Medicine, University of Sheffield, Sheffield, UK; 6 Laboratory of Biological Structure Mechanics (LaBS), Department of Chemistry, Materials and Chemical Engineering “Giulio Natta”, Politecnico di Milano, Milan, Italy; 7 PoliToBIOMed Lab, Department of Mechanical and Aerospace Engineering, Politecnico di Torino, Turin, Italy

**Keywords:** *In silico* modelling, Restenosis, Model validation, Multiscale modelling

## Abstract

**Background:**

Coronary artery restenosis is an important side effect of percutaneous coronary intervention. Computational models can be used to better understand this process. We report on an approach for validation of an *in silico* 3D model of in-stent restenosis in porcine coronary arteries and illustrate this approach by comparing the modelling results to *in vivo* data for 14 and 28 days post-stenting.

**Methods:**

This multiscale model includes single-scale models for stent deployment, blood flow and tissue growth in the stented vessel, including smooth muscle cell (SMC) proliferation and extracellular matrix (ECM) production. The validation procedure uses data from porcine *in vivo* experiments, by simulating stent deployment using stent geometry obtained from micro computed tomography (micro-CT) of the stented vessel and directly comparing the simulation results of neointimal growth to histological sections taken at the same locations.

**Results:**

Metrics for comparison are per-strut neointimal thickness and per-section neointimal area. The neointimal area predicted by the model demonstrates a good agreement with the detailed experimental data. For 14 days post-stenting the relative neointimal area, averaged over all vessel sections considered, was 20 ± 3% *in vivo* and 22 ± 4% *in silico*. For 28 days, the area was 42 ± 3% *in vivo* and 41 ± 3% *in silico*.

**Conclusions:**

The approach presented here provides a very detailed, location-specific, validation methodology for *in silico* restenosis models. The model was able to closely match both histology datasets with a single set of parameters. Good agreement was obtained for both the overall amount of neointima produced and the local distribution. It should be noted that including vessel curvature and ECM production in the model was paramount to obtain a good agreement with the experimental data.

**Electronic supplementary material:**

The online version of this article (10.1007/s13239-019-00431-4) contains supplementary material, which is available to authorized users.

## Introduction

Coronary artery disease is one of the most widespread causes of mortality in industrialized countries.^39^ Coronary artery stenosis, or abnormal narrowing, can lead to ischemia and potentially fatal heart attacks. This narrowing is often corrected by deploying a stent in the affected artery to keep it open and maintain blood flow.^23^,^24^ Currently, there are multiple types of stents in use, ranging from simple bare metal stents (BMS) to drug eluting stents (DES), and bioresorbable vascular scaffolds (BVS).^46^ Other advanced designs, such as stents that capture endothelial progenitor cells, are also being considered.^52^

During the stenting procedure, the narrowed artery is damaged by the stent struts being pressed into the vessel wall, as well as by the expanding balloon which is used to distend the artery and deploy the stent. This in turn causes a healing response in the vessel wall, which, if it becomes excessive, can cause a new narrowing of the vessel, or in-stent restenosis (ISR).^25^,^26^ In 5 to 10% cases ISR requires a repeat revascularization of the target lesion.^17^ Since it is formed by growth and proliferation of smooth muscle cells (SMCs) in the vessel wall, the composition of a restenotic lesion is different from the initial lesion: a restenotic lesion mainly consists of SMCs and the extracellular matrix (ECM) they produce.^8^,^15^,^31^ The initial lesion, on the other hand, usually consists of low-density lipoprotein (LDL), monocytes, macrophages, fat-laden foam cells and necrotic debris accumulated in an inflamed region in the presence of disturbed flow.^12^,^18^,^42^

ISR is associated with excessive damage to the vessel wall and with disturbed flow patterns in the stented vessel, in particular with low values of wall shear stress (WSS).^26^,^28^,^33^,^45^ Since ISR is an important complication of stenting, which can lead to various comorbidities and reduced quality of life, it is studied clinically (reviewed in Ref. 20), as well as in various *in vivo* (reviewed in Ref. 22), *in vitro*^3^,^19^ and *in silico*^5^,^6^,^16^,^30^,^35^,^41^,^48^,^55^^–^57 models. Computational models of ISR usually represent cells by on-lattice or freely moving agents, but continuum-based models have also been proposed.

Several reports in the literature have focussed on the formulation of the modelling approach applied to highly idealized arterial and stent geometries. Keshavarzian *et al*.^30^ coupled a 3D on-lattice agent-based model (ABM) to a finite element method (FEM) model to calculate the strain and stress in the tissue. Zahedmanesh *et al*.^56^ used a 2D FEM model of stent deployment coupled with an agent-based model of SMC proliferation and extracellular matrix (ECM) generation (also 2D). Nolan and Lally^41^ modelled growth in an off-lattice agent-based model in a 2D circumferential section of a stented artery. Fereidoonnezhad *et al*.^16^ proposed a purely FEM formulation of restenosis after angioplasty. Li *et al*.^35^ described a fully coupled 2D ABM-FEM framework that bi-directionally links finite-element stress calculations to the changing cell geometry.

In the idealised approaches described above the artery geometry is usually assumed to be cylindrical, and either straight longitudinal segments or circular cross-sections are considered in 2D. Since a realistic geometry is not used, predicted outcomes from these models have been compared to experimental data at the whole-artery level, using averaged neointimal area and similar metrics. A popular source of experimental data is a paper by Schwartz *et al*. from 1996^44^ (used e.g. in Refs. 35, 41 and 57), which reports the average amount of lumen loss in restenotic arteries at various time points after stenting. However, many models predict neointimal formation exclusively around the struts. This does not agree well with the experimental data, which shows a more even neointima.^1^,^4^,^22^,^32^ To validate the models on a location-specific level, and to make predictions about restenosis development in real vessels, a more realistic arterial geometry has to be considered, ideally comparing restenosis progression *in vivo* and *in silico* in exactly the same arteries.

In earlier work we developed an *in silico* model for in-stent restenosis. The initial version was two-dimensional,^48^^–^50 which was later extended into three dimensions to better replicate *in vivo* growth dynamics and to enable more realistic stent geometries.^57^ In this work, the model is extended to include ECM production and a modified mechanical model for internal and external elastic laminae (IEL and EEL). The EEL separates the middle SMC-rich vessel wall layer, tunica media, from the outer layer, tunica adventitia. The IEL lines the inner surface of the vessel and separates tunica media from the innermost layer of the vessel wall, tunica intima, which is composed of endothelial cells. Additionally, in this version of the model arterial curvatures are taken into account.

The model introduced in Ref. 57 did not include ECM production. In that version of the model, neointimal (NI) growth was on average about 1.5 times lower than the *in vivo* results for similar injury scores. Since experimental studies report that the neointima includes around 50–80% of ECM by volume,^15^,^18^,^29^ ECM production was therefore included in the model, which is described below.

Experimental evidence suggests that neointimal tissue is heterogeneous with morphologically distinct regions observed in human peripheral restenotic lesions. One type of tissue is composed of loose connective tissue with wide spaces between cells, and the second type is composed of dense connective tissue with cells closer together.^53^ This study also found that zones of loose ECM are rich in proteoglycans, in particular versican, which is involved in trapping water in extracellular tissue together with hyaluronan. Furthermore, this study found that the regions of dense ECM are rich in collagen and either contain tightly packed elongated SMCs or resemble fibrous plaques.

Other studies suggested mechanical deformation, such as low amplitude biaxial strain, as one of the mechanisms for increased versican production.^34^,^54^ There is also evidence that low flow promotes proteoglycan production.^29^ In particular, however,^34^ reports 4% strain as sufficient for production of versican-rich ECM.

Also, SMCs are not uniformly distributed in the neointima from lumen to media. Histology shows that there is a higher concentration of SMCs near the lumen, while large areas of loose ECM with few SMCs are found away from the lumen.^10^,^13^,^31^ The reason for this heterogeneity is considered to be SMC migration, or chemotaxis, along the gradient of growth factor concentration.^30^ Growth factor concentration is the highest near the lumen, so that is where synthetic SMCs tend to concentrate.^51^ These studies serve as a basis for the ECM model, described in detail in the next section.

The extended model of in-stent restenosis is then applied to stent geometries based on micro-CT scans of stents deployed in two porcine coronary arteries, including the curvature of the vessel, which more closely represents *in vivo* coronary vessels and results in significant secondary flows and helical flow patterns. The results obtained for these stent deployments are then compared to detailed location-specific *in vivo* histological growth data obtained for the same stent geometries.^38^

## Materials and Methods

### General Description of the ISR Multiscale Model

In-stent restenosis is a complex process influenced by factors acting on different scales. To capture these processes, a fully coupled multiscale model of ISR was developed. The model is described in detail in a previous publication.^57^ Here a brief overview is provided, with focus on the additions made to the model.

The blood flow through the stented segment is explicitly modelled, as well as growth, proliferation and movement of SMCs, their interaction with the IEL and EEL, and ECM production. For regeneration of endothelial cells (ECs) an implicit model is used. All these submodels communicate relevant data to each other at every iteration of the agent-based growth and proliferation model, which operates at the largest timescale of all the single-scale models, and therefore has the largest time step.

**Figure 1:**
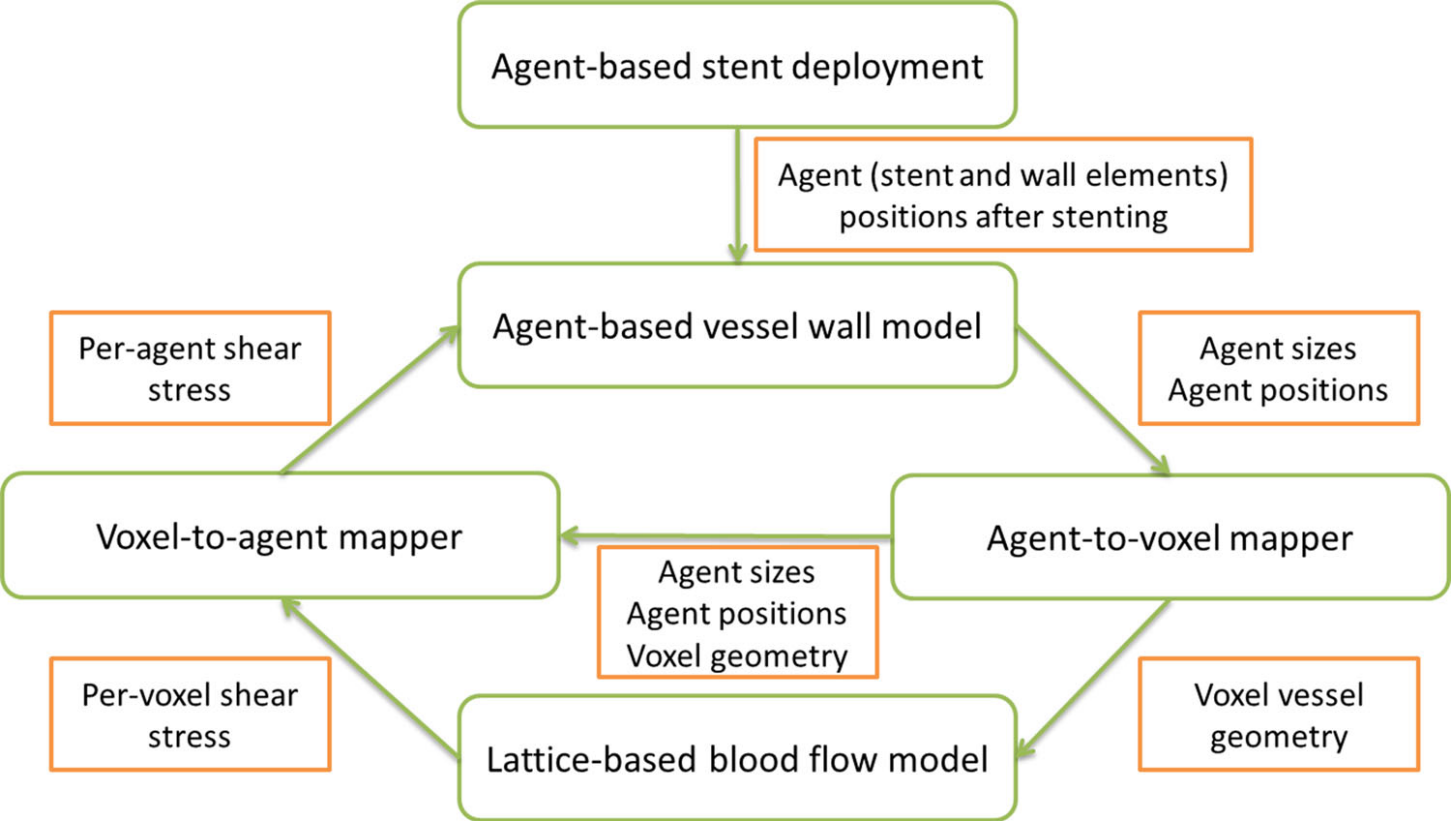
An overview of the multiscale in-stent restenosis model, illustrating data flow between single-scale components. After simulating stent deployment, post-stenting geometry is used as a starting point for the off-lattice agent-based simulation of neointima formation. From agent sizes and positions, a voxel-based geometry of the vessel is constructed, which is used to calculate flow in the vessel. The flow produces shear stress on the vessel wall, which is mapped back to the agent-based model to inform the next iteration of neointima formation simulation. For more detail, see also Ref. 57

The relationship between the main submodels is shown in Fig. 1: (i) agent-based stent deployment, which captures the mechanical deformation of the vessel wall during stent deployment and damage to the wall caused by excessive mechanical stress and strain; (ii) an agent-based model for restenotic processes in the wall, which covers SMC growth and proliferation, ECM production, EC regeneration, and also keeps all arterial wall components in a state of mechanical equilibrium; (iii) a blood flow model, which calculates the flow inside the stented vessel.

The blood flow model uses a Lattice Boltzmann formulation to calculate the flow in the channel.^47^ The lattice for the blood flow model is constructed by mapping the agents from the agent-based model to voxels. Additionally, the agent-to-voxel mapper passes the constructed mapping to the voxel-to-agent mapper, which uses it to calculate per-agent WSS from voxel-based flow calculated in the blood flow model. The agents that make up the vessel wall are modelled mechanically as elastic spheres connected by springs, and these agents also have a biological ruleset that governs the cell growth and proliferation. The SMCs switch to a synthetic phenotype when they are exposed to the lumen immediately post-stenting. The growth and proliferation of SMCs are suppressed if the cell is contact inhibited or if the local concentration of nitric oxide, produced by the ECs, is sufficiently high. The stent deployment is performed by radially expanding the stent from the vessel centreline to a predetermined final shape. For time integration of movement, a variable-step 4th order Runge–Kutta solver is used.

For the simulations described in this paper, the final stent shape is reconstructed from *in vivo* post-stenting micro-CT data. The detailed description of the model formulation, as well as references to the experimental works used to calibrate the model parameters, can be found in our previous publication.^57^ Unless specifically noted, the parameters of the model used here are similar to the ones used in Ref. 57. Supplementary Table 1 includes a list of biological parameters used in the model, as well as their source publications.

### Experimental *In Vivo* Data

The experimental *in vivo* data used to validate the model were obtained by stenting healthy porcine coronary vessels with oversized balloons. The *in vivo* experiments were performed as a part of an earlier study, and the experimental protocol has been reported previously.^38^ In brief, the stents were deployed in right coronary arteries (RCA) by using over-inflated balloons to induce restenosis (approx. 1.4:1 balloon-to-artery ratio). The stents were harvested 14 and 28 days post-stenting, high-resolution micro-CT scans were obtained, and three-dimensional (3D) surface meshes of the stents were reconstructed from this data. Following micro-CT, the stented vessels were embedded in methacrylate resin T8100 (TAAB Laboratories), sectioned, and submitted for histology. The *in vivo* vessels were fixed in resin, slide-mounted and cut with a high-speed precision saw. The cross-sections are spaced 1 mm apart, which gives a rough location along the stent. The corresponding *in silico* cross-section is then found by using stent struts as landmarks. The sections were parallel and vertical to the line connecting the proximal and distal ends of the stents. Full details on the sectioning procedure can be found in Ref. 36.

### Simulation Procedure

The simulation procedure is as follows: first, agent-based stent geometries were generated by filling the meshes obtained from micro-CT with agents representing small volumes (30 × 30 × 30 *μ*m) of stent material. Then, an agent-based stent deployment model was used to expand the agent-based stent from inside the lumen into its original shape, similar to Ref. 57. The deployment occurs by radially expanding the stent into its original shape and equilibrating the inter-agent forces after the expansion is finished.

Then, the ISR simulation was executed for the resulting configuration for a set period of simulated time. Based on the spatial positions of agents, a voxel-based geometry of the vessel was constructed at each iteration of the biological growth model (1 h of simulated biological time). The flow in the artery is updated at each step based on the changing arterial wall geometry, and the relevant values, such as WSS distribution, are mapped back to the agents in the vessel wall.

Based on the experimental studies outlined in the introduction, we propose to ignore the volume of collagen-rich ECM for the purpose of restenosis progression, and assume the SMCs in collagen-rich regions to be densely packed. For loose proteoglycan-rich ECM, the following production model was implemented: strained synthetic SMCs (> 10% mechanical strain, selected based on the upper bound for production of versican-rich ECM in Ref. 34) and their daughter cells produce loose proteoglycan-rich ECM at a constant rate until they switch back to the contractile phenotype. The ECM is produced by these cells in blobs, represented as agents with a volume equal to that of a contractile SMC. The ECM agents have the same mechanical interaction rules as the SMC agents,^57^ and also the interaction between SMC and ECM agents follows the same rules. The ECM blobs are produced by the strained synthetic SMCs stochastically to avoid stepwise growth (large instantaneous increases in neointimal area).

Stepwise growth would happen if, for example, the ECM agents were produced on a timer, since for two daughter cells of a single SMC the ECM production timer would start simultaneously. Two problems with stepwise growth are that it is unphysiological, and also adding many neighbouring agents at a single step would create large artificial stresses in the neointima.

**Figure 2:**
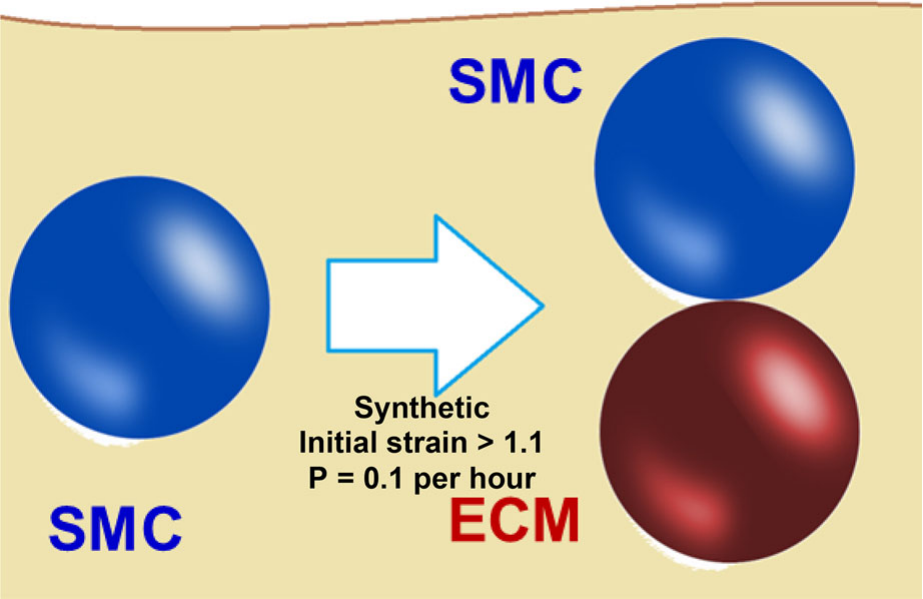
Schematic of freshly produced loose ECM blob placement in the model (vessel lumen at the top); ECM is placed away from the lumen to mimic SMC migration.

The stochastic model was chosen based on the neointimal composition values of 50–80% ECM by volume reported in the literature.^15^,^18^,^29^ The probability of ECM blob production is set to *P* = 0.1 per hour per SMC; on average this results in 3 blobs of ECM produced during a cell cycle, and consequently in 60% of the local tissue being loose ECM, which is in line with experimental estimations. The produced ECM blobs are placed away from the lumen, relatively to the producing SMC agent, to mimic smooth muscle cell migration by chemotaxis, pushing the producing cell towards the lumen (schematically illustrated in Fig. 2).

Also, the mechanical parameters of the IEL and EEL have been adjusted as compared to the earlier model.^57^ In this version, a mechanical difference is introduced between IEL-covered and bare SMCs. Using this approach, when IEL ruptures from excessive strain and some IEL-covered SMC agents are replaced with regular SMC agents (with weaker interaction strength), the remaining IEL-covered agents retract from the rupture and reduce the strain in the intact areas of IEL. Note that the strain, unlike stress, does not directly depend on the mechanical properties of the IEL, and the threshold value for IEL strain has been directly characterised from experiments.^21^ The same approach is applied to the EEL as well, with the exception that it is only broken in the most extreme cases. Lamina-covered SMCs in this version of the model have two times the attraction force between themselves compared to non-lamina-covered, which in case of lamina rupture allows the tissue to pull back and reduce local mechanical strain. This addition reflects the tensile strength added by the IEL and the fact that when the IEL breaks, the local tensile strength diminishes. The ratio of tensile strength between lamina vs. no lamina covered SMCs is arbitrary as the authors were unable to find comparisons between IEL and a single layer of SMCs in terms of strength. Since in this model we are not interested in the fine details of arterial mechanics, this difference is sufficient to reflect the *in vivo* phenomena: the broken IEL retracts, instead of staying in the same place, and this exposes medial SMCs to the lumen and promotes growth.

Additionally, the vessel geometry was modified to reflect the curved shape of *in vivo* coronary vessels. In these vessels curvature results in a significant flow helicity,^7^ which in turn affects the local WSS. Since WSS indirectly controls SMC proliferation and is an important parameter in the model,^40^ the curvature was added to the model geometry. Vessels were approximated with toroidal segments, with Poiseuille flow at the inlet, and a zero-gradient boundary condition on the outlet. To avoid unphysiological axial strain in the vessel wall pre-stenting, the radii of the agents were scaled based on their position relative to the outer and inner curvatures, so that in a curved vessel segment the agents were initially in equilibrium. The vessels’s centerline is assumed to be a plane curve, positioned according to the visible residual curvature in the micro-CT images of stents.

Stents were deployed in these segments using a procedure similar to the one described in Ref. 57 for straight vessels. The radius of curvature of the toroidal segments was chosen as 28 mm. This value is in the middle of the curvature range used in an earlier publication concerning flow assessment in the same stent,^7^ where the range of curvatures was obtained by a 2D spline approximation of the centroid path in the RCA in three similar porcine models. Also, the range was assessed to be reasonably within measurements from single-plane coronary angiograms of the human RCA.^37^ It is worth noting that in Ref. 7, the most pronounced differences in flow patterns were observed between the straight and curved vessels, not between different curvatures. Additional simulations were performed for straight vessels of similar diameter, and their results were compared to the results obtained for a curved geometry.

One simulation run was performed for each considered stent. Due to the extremely large number of agents (more than 5 million), inherent stochasticity in the SMC model has only a minor effect on the average output values.^57^ Therefore, one run for each simulation is considered sufficient. The simulations were performed using a 32-core Intel Xeon E5-2650 (2.00 GHz) server. The 14-day simulation took about 25 h of (wall) computational time, and the 28-day simulation took about 42 h.

### Location-Specific Comparison

For each *in vivo* stent (explanted after 14 and 28 days respectively), four histological sections were chosen, spaced from the proximal to distal ends of the stent. Since the neointimal growth is relatively smooth, neointimal thickness for neighbouring points is strongly correlated. Because of this, analysing additional slices located close to the existing ones does not add much information about the neointimal growth. Particular slides that had relatively few (< 10) stent struts embedded were selected for ease of landmarking.

For each histological section, a corresponding section from the simulated stented vessel was taken at the same location. For each pair of sections, corresponding struts were identified *in vivo* and *in silico* and the neointimal thickness at each strut was measured.

Furthermore, we also measured the total NI area for each slide. Following Refs. 7 and 27 NI area was defined as the difference between the area enclosed by the stent struts and the lumen area. Note that this is not the true area of the neointima, since the vessel wall does not form a straight line between struts after stenting. However, this metric uses discrete and easily identifiable reference points for the outer border (stent strut centers) instead of the border between the neointima and the media, which is harder to identify visually and can introduce additional errors. Hence, this measurement can be defined unambiguously based on clearly visible points (struts and wall-lumen border), without relying on visually identifying the boundary between the original media and the neointima. This means that, for comparison of the lumen loss *in vivo* and *in silico*, this definition results in a well-defined metric.

## Results

### Stent Deployment

**Figure 3:**
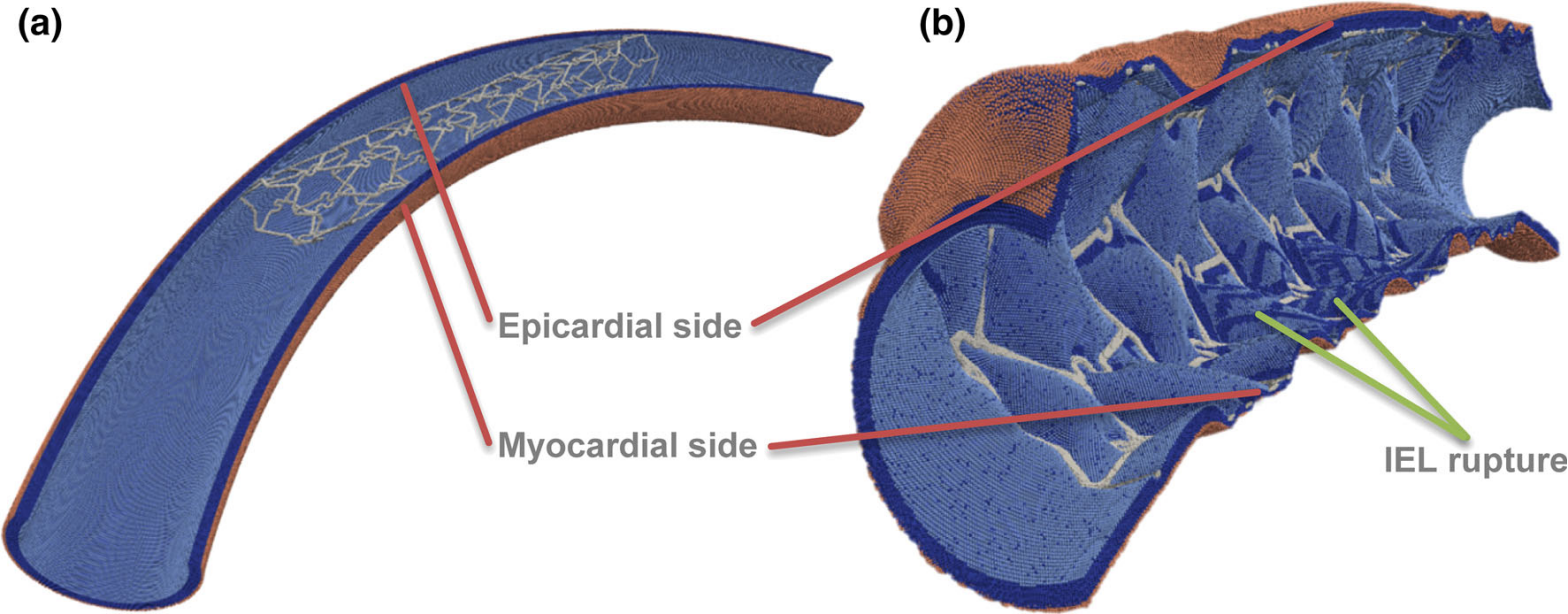
Toroidal vessel segment (a) pre- and (b) post-deployment. IEL ruptures are visible on the inner curve, as well as near some struts. SMCs—dark blue, IEL—light blue, EEL—beige, stent—light grey.

Stents were deployed in the toroidal vessel segments. Due to the vessels being more curved pre-stenting than the observed *in vivo* curvature of the stents, some hinge-like local bending effects were observed at the stent ends (Fig. 3). The deployment resulted in an uneven strain on the epicardial (outer curve) and myocardial (inner curve) sides of the vessels. The larger strain on the inner curve resulted in ruptures in the IEL during stent deployment and to heightened strain in the tunica media, which consequently led to increased loose ECM production relative to the outer curve, according to the rules previously described.

**Figure 4:**
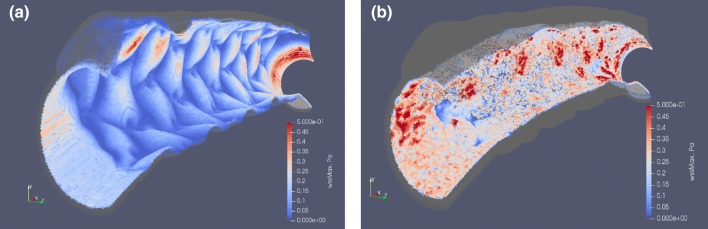
Simulated vessel wall, colored by local WSS: (a) immediately post-stenting; (b) 28 days post-stenting. Areas of high WSS sufficient for growth arrest in our model are shown in red, areas of low WSS are in blue. Note that the growth arrest only happens in the presence of functional endothelium, and after growth arrest the cells remain quiescent even if WSS decreases later.

Furthermore, similar to published results,^7^ the WSS was uneven on the epicardial and myocardial sides, with larger areas of low WSS on the myocardial side (Fig. 4a). Areas of high WSS sufficient for growth arrest in our model are shown in red, areas of low WSS are in blue. The growth arrest happens in the model in the presence of functional endothelium and WSS > 0.27 Pa.

### Neointimal Growth

**Figure 5:**
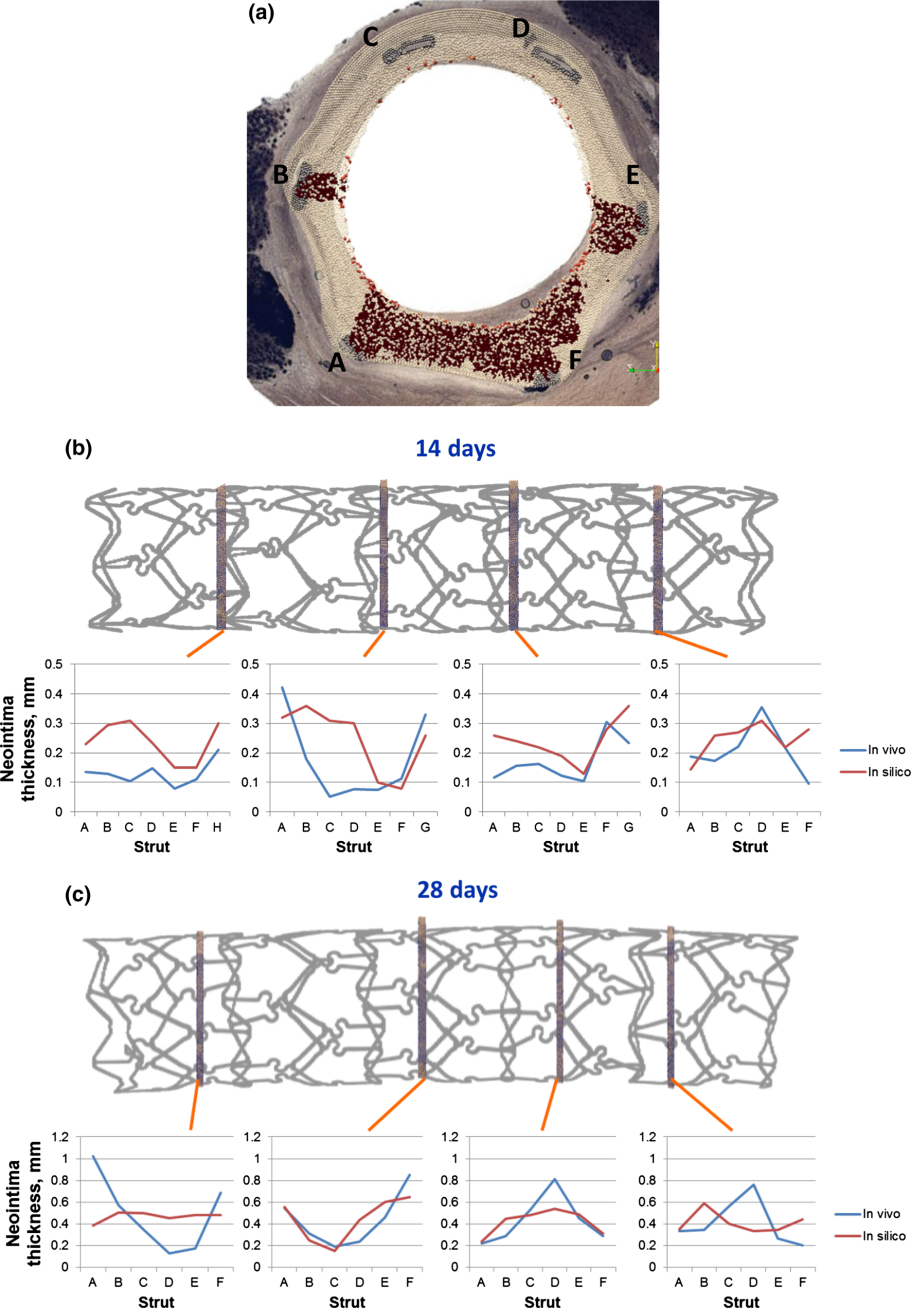
(a) superposition of a simulated and an experimental slice of artery taken from the middle part of the 28-day vessel, areas of loose ECM in the simulated slice are in dark red, letters denote stent strut IDs; (b, c) positions and extent of per-strut growth *in vivo* and *in silico* for 14 and 28 days post-stenting. Struts are assigned sequential IDs (A–H) for each slice, and neointimal thickness was measured from the middle of each strut along the line toward the center of the lumen. Proximal end on the left. Strut G on the proximal slice of 14-day stent was excluded from analysis, since it was not present on the *in silico* slice, possibly due to stent deformation between micro-CT and sectioning.

**Figure 6:**
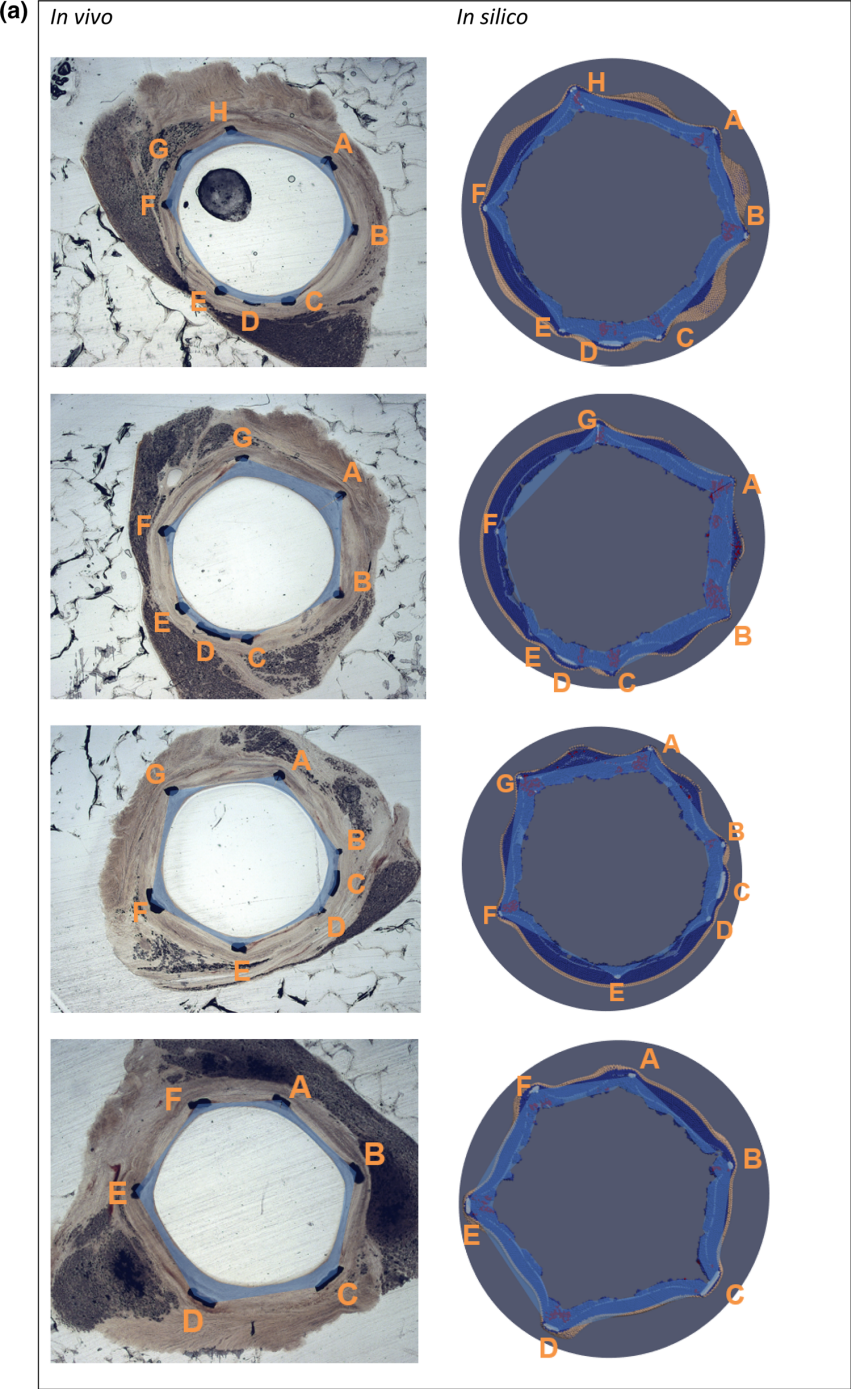

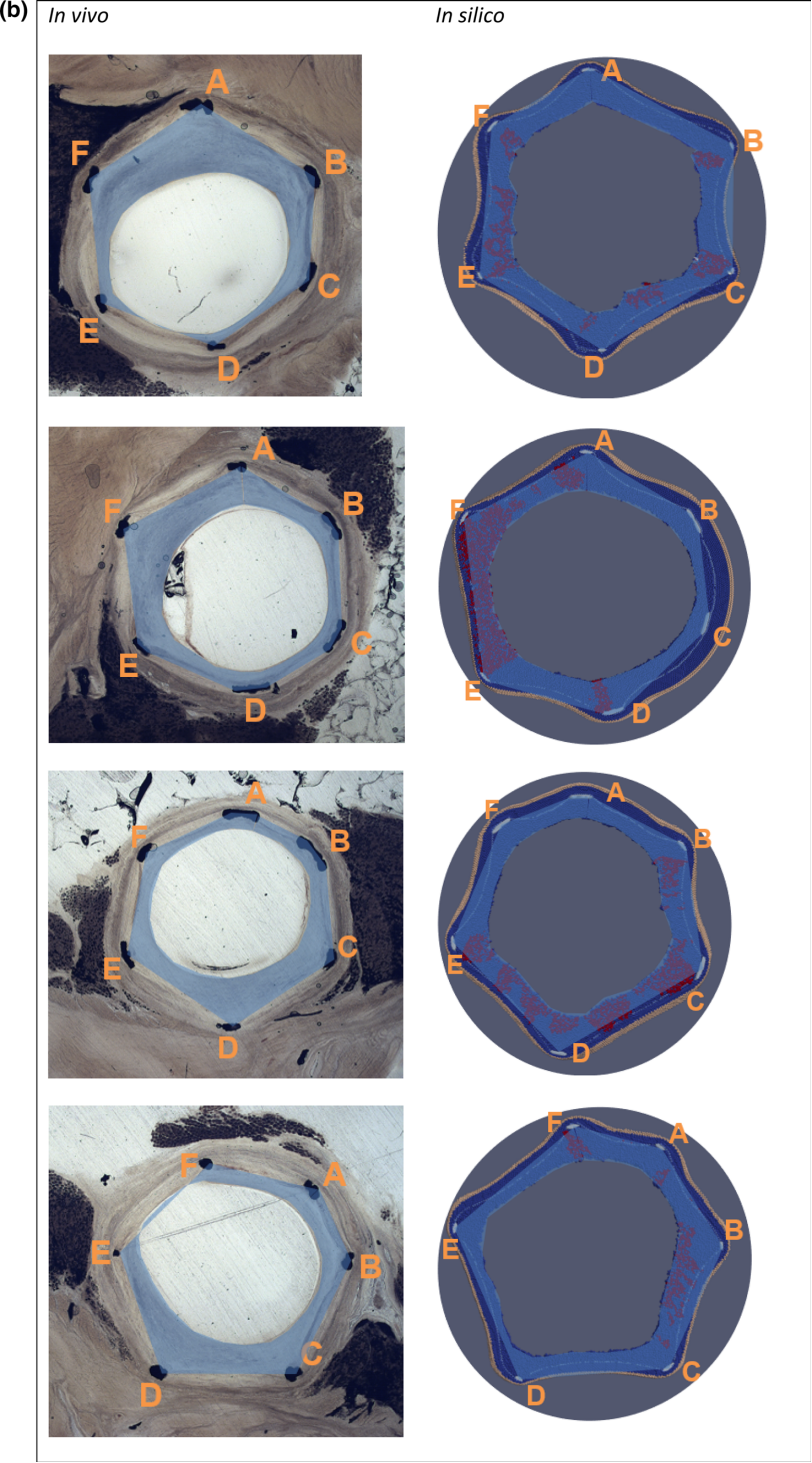
Vessel slices *in vivo* and *in silico*. *In silico* slices are rotated to match the orientation of *in vivo* slides. Stent explanted (a) 14 days; (b) 28 days post-stenting. Slides order from proximal to distal. Letters denote strut IDs, see Fig. 5. Blue areas show neointima estimation, as described in the text. If the lumen is outside the area enclosed by the struts (see e.g., EF in the 1st *in vivo* slide), the area is taken as negative. *In silico* images: SMCs—dark blue, IEL—light blue, EEL—beige, ECM—red, stent—light grey.

After simulating post-stenting neointimal growth over 28 days, the vessel lumen becomes smoother (Fig. 4b). Growth stops almost completely at this point in the model, which is similar to the results observed in pigs *in vivo.*^1^ Figure 5a shows a superposition of a simulated and histological section from the middle part of the 28-day stent. Simulated and histological images for other sections can be found in Fig. 6.

Figures 5b and 5c shows the measured NI thickness for stents explanted after 14 and 28 days respectively. Struts were assigned sequential IDs (A–H) for each slice, and NI thickness was measured from the middle of each strut along the line toward the center of the lumen. Strut G on the proximal slice of the 14-day stent was excluded from analysis, since it was not present on the *in silico* slice, possibly due to stent deformation between micro-CT and sectioning.

**Figure 7:**
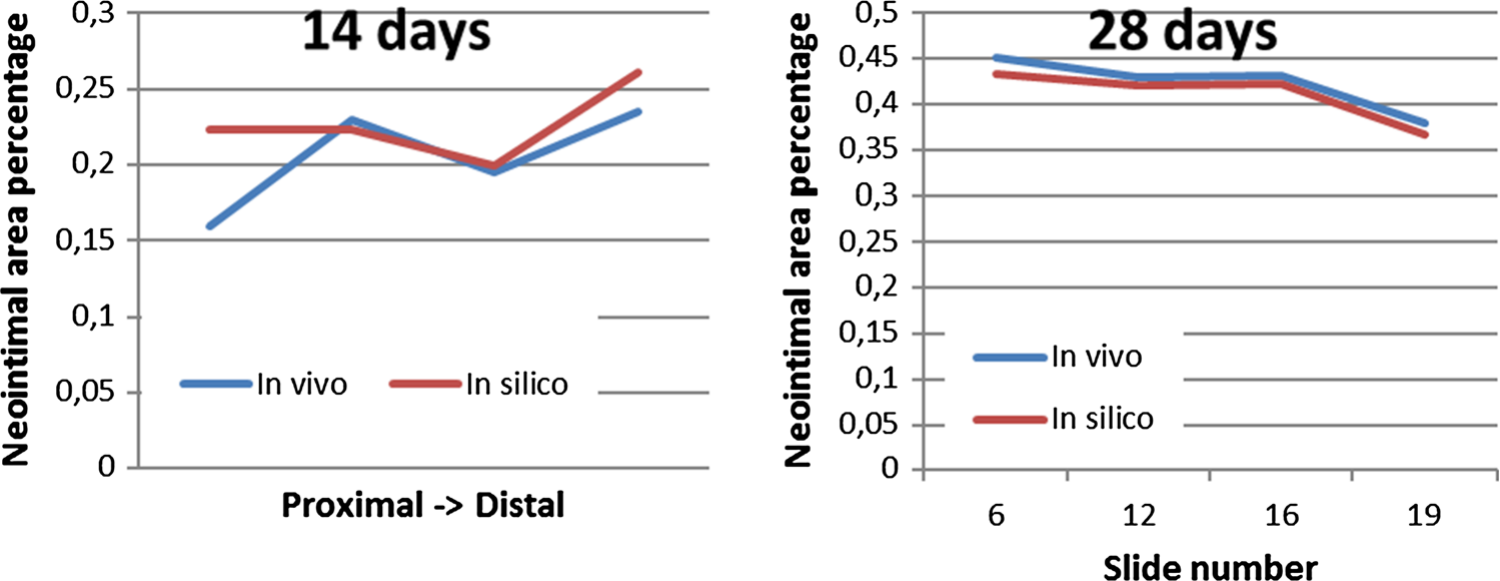
Relative NI area for 14 and 28 days post-stenting, for *in vivo* and *in silico* data. Measurements were taken at the locations shown in Fig. 6.

**Figure 8:**
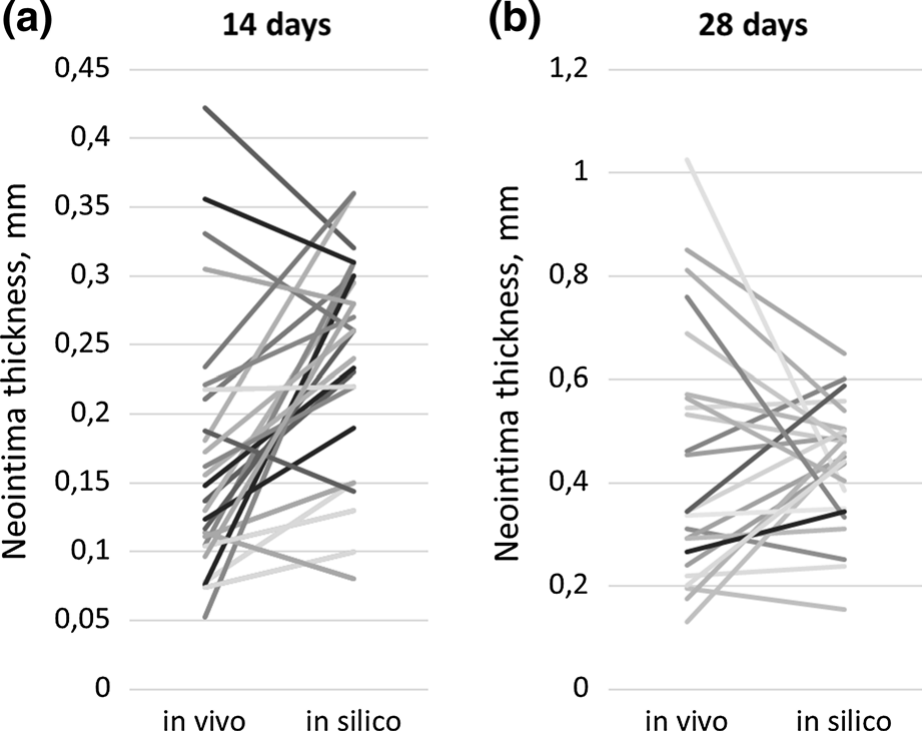
Paired dots plot for 14 (a) and 28 (b) days post-stenting, for *in vivo* and *in silico* data. Measurements were taken at the locations shown in Fig. 6.

NI area was then measured for the same set of slides shown on Figs. 5b and 5c. Neointimal areas for each *in vivo* and *in silico* slice are shown in Fig. 6. Relative NI area was calculated as the ratio between NI area and the area enclosed by the struts. The relative NI area is shown in Fig. 7. Figure 8 shows a paired dots plot for 14 (a) and 28 (b) days post-stenting, for *in vivo* and *in silico* data.

### Curved and Straight Vessel Geometries Result in Different Growth Patterns

**Figure 9:**
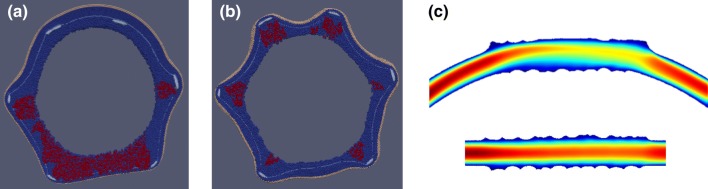
(a, b) The extent of growth in a curved (a) and cylindrical (b) geometries for the 2nd slide of the 28-day stent, epicardial side on top. Cylindrical vessel approximation results in loose ECM deposits around each strut, as well as in strong protrusions on the epicardial side of the vessel, which are not observed *in vivo*. (c) flow velocity magnitude in the middle longitudinal section of both vessels.

In addition to the curved vessel described above, the model was also applied to a similar stent in a straight cylindrical vessel, similar to a previous publication.^57^ Figures 9a and 9b shows the extent of growth in a curved (a) and cylindrical (b) geometries for the 2nd slide of the 28-day stent, epicardial side on top. The cylindrical vessel approximation results in loose ECM deposits around each strut, as well as in strong protrusions on the epicardial side of the vessel, which are not observed *in vivo*. Figure 9c shows the flow velocity magnitude in the middle longitudinal section of both vessels. The flow in the curved vessel creates areas of high WSS on the top side and of low WSS on the bottom side.

## Discussion

The updated model for in-stent restenosis demonstrates good agreement with *in vivo* experimental histology data on a per-strut basis and matches the *in vivo* neointimal area closely. To the best of our knowledge this is the first attempt to compare a complex multiscale model of in-stent restenosis with histological data at such a detailed local level. This detailed comparison allows for location-specific comparisons between *in vivo* and *in silico*, which enables a much richer validation process.

Neointimal area (and correspondingly, the patent lumen diameter) is, together with stenosis length, one of the most important factors for hydrodynamic resistance and physiological significance of a restenosis. The total extent of growth in the simulation is similar to the *in vivo* data, even though the detailed local distribution of growth differs. Also, as well as in our previous model,^57^ neointimal growth can be observed far away from the struts, similarly to experimental data.^1^,^4^,^22^,^32^

Many earlier restenosis models, on the other hand, predict a very focal growth around the struts.^5^,^35^,^41^,^48^,^56^ This may be caused by the assumption made in these models that the growth originates only from the places where the stent contacts with the artery, while in our model it also originates from rupture of the strained IEL far away from the stent struts. Another assumption that is often made is that endothelial layer is only disturbed locally near stent struts. This does not agree with the experimental data of Rogers *et al*.^43^ who report that in en face photomicrographs of stented arteries there are large areas of exposed media far away from the struts, while close to the struts areas of intact endothelium can be found, presumably where the balloon didn’t come into contact with the artery wall during stent deployment.

The nonuniform growth in the model reported here is caused by several factors. The first one is the difference in WSS in different locations, which affects the growth inhibition. Two other factors are related to the damage done to the wall, which is nonuniform because the stent curvature is different from the unstented vessel curvature. First, this nonuniform damage causes tears in the internal elastic lamina, which cause larger initial proliferation. Second, it also affects the initial strain of the medial SMCs, which in our model leads to an increased production of loose ECM.

The observed differences in per-strut thickness can be in part due to the difference in *in vivo* and reconstructed vessel curvature radius and direction, which affects the simulated damage, strain and WSS. It should be noted that the curvature isn’t based on the exact same animal, since that data was unavailable, but on the average of three similar adolescent porcine models. Additionally, a less good agreement in the neointimal area estimation at the stent ends compared to the middle section can be seen. This might be related to an inaccurate representation of the post-stenting geometry, caused by stent deployment effects, such as focal bending and scratching of the vessel or dogboning of the stent during the deployment, since those effects are currently not well represented in the model.

Furthermore, it is worth mentioning that both the addition of vessel curvature and the ECM production do not, by themselves, significantly increase the computational costs of the model, while contributing significantly to increased accuracy of the predictions. In particular, the ECM model consists of only a few straightforward rules, which are only executed once at each time step of the biological model. To simulate a curved vessel, however, a larger region of the vessel is considered, compared to the straight vessel approximation, to let the flow develop before entering the stented region.

The model is able to predict the total extent of growth closely for the case of a stented healthy porcine artery. However, to apply the model to diseased human arteries, several modifications and additions have to be introduced. First, a model of an atherosclerotic plaque has to be added, to predict its deformation during stent deployment and the diseased vessel morphology during the healing period. The more complicated geometry of a diseased vessel would also require a more detailed stent deployment simulation. Second, the parameters of the model also have to be adjusted to human physiology, reflecting longer healing and reendothelialization times and different vessel morphology, as well as a critical assessment of all explicit and implicit assumptions in the model, which capture the complex biological responses in neointima formation.

Also, after validating the model for the BMS case, it is possible to extend it to incorporate state-of-the-art stents, for example DES or bioresorbable scaffolds. This would require extending the model with submodels for drug elution and for stent polymer degradation. Note that for the two dimensional version of the model results of drug eluting stents have already been reported.^50^

There are some mechanisms that affect the restenosis formation, but are not included in the ISR model. Arterial cyclic strain has been shown to have a strong impact on endothelial cell migration and proliferation, which play important roles in vascular healing (e.g., in the ISR model the nitric oxide produced by the ECs is the main stopping condition for SMC growth). However, in our model, endothelium recovery is approximated as a uniform and stochastic process, happening in the whole stented segment at once. In an earlier publication^49^ this has been shown to be a better approximation than recovery from proximal and distal ends of the stent, however it obviously does not capture the fine details of the process, and might also contribute to the difference in per-strut growth for *in vivo* and *in silico* scenarios.

In addition, cyclic strain amplitude has been shown been shown to affect the growth of vascular SMCs.^11^ SMCs’ response to both the average value of cyclic strain and to its amplitude were tested, and reducing the amplitude of cyclic strain from normal physiological values, while keeping the mean strain similar, promoted proliferation in the SMCs. This effect is not studied in our model in detail, since for the cells in the stented region the amplitude of cyclic strain is assumed to be rather similar.

Myocardial contraction also may affect the process in multiple ways, one of them is by affecting the transmural pressure gradient, which causes a response in ECs as well as in SMCs (reported e.g., in Ref. 14). Part of this response includes production of NO in response to interstitial flow, but the levels of NO produced in this way are several times lower than those produced by ECs in the response to blood shear stress. However, the biological adaptive response of the cells to myocardial contraction, as well as reduced cyclic strain in the stented vessel, might have a significant effect on post-stenting recovery and requires further study.

One other important direction is to quantify the effects of uncertainties in the model’s inputs on the simulation results (uncertainty quantification) and to assess the sensitivity of the parameters in the model on the simulation results (sensitivity analysis). This has already been done for a two-dimensional version of the model.^40^ Most uncertainty came from the uncertainty in the endothelium regeneration speed, followed by uncertainty in stent deployment depth (and consequently the extent of injury sustained by the vessel). The preliminary results for the 3D model support the hypothesis that these two parameters are also most important in 3D, but a more detailed uncertainty quantification and sensitivity analysis study is required.

So far, the current model was only applied to the case of a stent in a single vessel. The clinically relevant case of bifurcation stenting^2^ has not yet been considered. The modelled vessel geometry is also rather simplified, not accounting for any unevenness of the vessel surface pre-stenting. This can partially be alleviated by using intravascular imaging data (e.g., optical coherence tomography (OCT) or intravascular ultrasound (IVUS) data) to reconstruct the unstented vessel.^9^

The current ECM production model simplifies all details of the biology into a few generic rules. These are sufficient for this case. If however the conditions change—for example, in a more complicated geometry where concentration profiles of growth factors are different—these rules might produce non-physiological behaviour, and further validation of the ECM production model is required.

Finally, a limitation of this study is that each time point was represented by a single animal, while in a larger population the restenotic response is likely to vary, and for validation multiple animals have to be considered for each time point. The stents were deployed as part of a larger study where six animals were used.^38^ In each animal, both RCA and LAD were stented with similar stents. However, micro-CT and subsequent stent reconstruction was only performed for two animals, and in both cases RCA was studied. More *in vivo* data are required to validate this model before applying it in, for instance, stent design studies and/or *in silico* clinical trials.

## Conclusions

In this paper an approach for validation of an *in silico* 3D model of in-stent restenosis in porcine coronary arteries is presented and illustrated by comparing the modelling results to *in vivo* data for 14 and 28 days post-stenting. This model of ISR reported here is based on a previously published model 57 that has been extended with ECM production and applied to *in vivo* stent geometries reconstructed from micro-CT. A detailed location- and strut-specific comparison between the *in silico* and *in vivo* data was performed, in addition to comparing an integral metric of total neointimal area in each histological slice.

The model was able to closely match both datasets with one single set of parameters. It should be noted that including vessel curvature and ECM production in the model was paramount to obtain a good agreement with the experimental data. The procedure introduced here can enable a richer validation process for models of in-stent restenosis, compared to using only integral metrics such as the average neointimal area. Our future plans are to extend the model to diseased arteries, human physiology, and to incorporate modern stent designs (BMS and BVS).

## Electronic supplementary material

Below is the link to the electronic supplementary material.

Supplementary material 1 (DOCX 27 kb)
