# Supplementary material for: Location-Specific Comparison Between a 3D In-Stent Restenosis Model and Micro-CT and Histology Data from Porcine In Vivo Experiments
Source: Cardiovasc Eng Technol. 2019 Sep 17;10(4):568–82. doi: 10.1007/s13239-019-00431-4 (PMC6863796; doi:10.1007/s13239-019-00431-4)
Supplement: Supplementary file 1 — Supplementary material 1 (DOCX 27 kb) [file 13239_2019_431_MOESM1_ESM.docx]

## Supplementary table 1. Biological parameters used in the model

| # | Name | Value porcine | Comments |
| --- | --- | --- | --- |
| 1 | Inner diameter | 2.8 mm (Keller, 2012) | Porcine data is what is currently used in the simulation (Yorkshire minipig RCA); |
| 2 | Outer diameter | 3.3 mm (Keller, 2012) | Does not include adventitia |
| 3 | Wall thickness | 0.25 mm (Tahir, 2013) |  |
| 4 | Blood velocity | 0.15 m/s (Huo, Choy, Svendsen, Sinha, & Kassab, 2009) | Average velocity at the center of the vessel |
| 5 | Endothelium regeneration time | 14 days (Nakazawa et al., 2010; Van Beusekom, Ertaş, Sorop, Serruys, & Van Der Giessen, 2012)  Healthy vessels  See comment section for details | Porcine: 53±36% after 2 days, 95±2% after 5 days, 99±2% after 14 days – EC coverage  59±25% after 3 days, 96±7% after 14 days (data over struts, used in porcine model) – PECAM−1 (platelet endothelial cell adhesion molecule) aka CD31  Between struts: 81±14%, 100±0% respectively (Nakazawa et al., 2010).  Data for Genous stent was used instead of BMS. The reasoning was that the Genous stent accelerates reendothelization, but does not reduce the hyperplasia, hence it is assumed extra ECs are dysfunctional, and PECAM-1 expression is the same as BMS |
| 6 | Curvature radius | 28-36 mm (Keller, 2012)  Degree of the curve  ~39-51° for a 25 mm segment | Degree of curve:  30-66°, avg. 52°±9  Length:  10.2±1.7 cm (5.7-14.5 cm) (Messenger et al., 2000) |
| 7 | SMC cell cycle | 32 hours (Tahir, 2013) | Some SMCs in the cultures are likely inhibited; low doubling time requires lots of PDGF |
| 8 | SMC agent radius | 0.015 mm (Tahir, 2013; Zun, Anikina, Svitenkov, & Hoekstra, 2017) | Radius is an estimation (SMCs are not round in reality)  Probably the same radius is a good enough approximation |
| 9 | NO production rate | See (Guo & Kassab, 2009; Tahir, 2013) for NO in relation to WSS | For more detailed data see source; porcine data for pig hind limbs of varying size |
| 10 | NO growth inhibition threshold | 1000 nM (Coneski & Schoenfisch, 2012; Thomas et al., 2008) | In vitro cell cultures, murine and human mostly |

Coneski, P. N., & Schoenfisch, M. H. (2012). Nitric oxide release: Part III. Measurement and reporting. *Chemical Society Reviews*, *41*(10), 3753. https://doi.org/10.1039/c2cs15271a

Grainger, D., Kirschenlohr, H., Metcalfe, J., Weissberg, P., Wade, D., & Lawn, R. (1993). Proliferation of human smooth muscle cells promoted by lipoprotein(a). *Science*, *260*(5114), 1655–1658. https://doi.org/10.1126/science.8503012

Guo, X., & Kassab, G. S. (2009). Role of shear stress on nitrite and NOS protein content in different size conduit arteries of swine. *Acta Physiologica*, *197*(2), 99–106. https://doi.org/10.1111/j.1748-1716.2009.01999.x

Huo, Y., Choy, J. S., Svendsen, M., Sinha, A. K., & Kassab, G. S. (2009). Effects of vessel compliance on flow pattern in porcine epicardial right coronary arterial tree. *Journal of Biomechanics*, *42*(5), 594–602. https://doi.org/10.1016/j.jbiomech.2008.12.011

Keller, B. K. (2012). In-stent restenosis and coronary curvature: Translational approach to computational fluid dynamics.

Messenger, J. C., Chen, S. Y. J., Carroll, J. D., Burchenal, J. E. B., Kioussopoulos, K., & Groves, B. M. (2000). 3D coronary reconstruction from routine single-plane coronary angiograms: Clinical validation and quantitative analysis of the right coronary artery in 100 patients. *International Journal of Cardiac Imaging*, *16*(6), 413–427. https://doi.org/10.1023/A:1010643426720

Nakazawa, G., Granada, J. F., Alviar, C. L., Tellez, A., Kaluza, G. L., Guilhermier, M. Y., … Virmani, R. (2010). Anti-CD34 Antibodies Immobilized on the Surface of Sirolimus-Eluting Stents Enhance Stent Endothelialization. *JACC: Cardiovascular Interventions*, *3*(1), 68–75. https://doi.org/10.1016/j.jcin.2009.09.015

Tahir, H. (2013). *Modelling and Simulating the Dynamics of In-Stent Restenosis in Porcine Coronary Arteries*. PhD thesis, UvA.

Tanner, F. C., Meier, P., Greutert, H., Champion, C., Nabel, E. G., & Lüscher, T. F. (2000). Nitric Oxide Modulates Expression of Cell Cycle Regulatory Proteins. *Circulation*, *101*(16), 1982–1989. https://doi.org/10.1161/01.cir.101.16.1982

Tantini, B., Manes, A., Fiumana, E., Pignatti, C., Guarnieri, C., Zannoli, R., … Galié, N. (2005). Antiproliferative effect of sildenafil on human pulmonary artery smooth muscle cells. *Basic Research in Cardiology*, *100*(2), 131–138. https://doi.org/10.1007/s00395-004-0504-5

Thomas, D. D., Ridnour, L. A., Isenberg, J. S., Flores-Santana, W., Switzer, C. H., Donzellie, S., … Wink, D. A. (2008). The chemical biology of nitric oxide. Implications in celular signaling. *Free Radical Biology and Medicine*, *45*(1), 18–31. https://doi.org/10.1016/j.freeradbiomed.2008.03.020.The

Van Beusekom, H. M. M., Ertaş, G., Sorop, O., Serruys, P. W., & Van Der Giessen, W. J. (2012). The Genous^TM^ endothelial progenitor cell capture stent accelerates stent re-endothelialization but does not affect intimal hyperplasia in porcine coronary arteries. *Catheterization and Cardiovascular Interventions*, *79*(2), 231–242. https://doi.org/10.1002/ccd.22928

Zun, P. S., Anikina, T., Svitenkov, A., & Hoekstra, A. G. (2017). A Comparison of Fully-Coupled 3D In-Stent Restenosis Simulations to In-vivo Data. *Frontiers in Physiology*, *8*(May), 284. https://doi.org/10.3389/fphys.2017.00284
